# A Fur-like protein PerR regulates two oxidative stress response related operons *dpr* and *metQIN* in *Streptococcus suis*

**DOI:** 10.1186/1471-2180-12-85

**Published:** 2012-05-30

**Authors:** Tengfei Zhang, Yi Ding, Tingting Li, Yun Wan, Wei Li, Huanchun Chen, Rui Zhou

**Affiliations:** 1 Division of Animal Infectious Diseases in the State Key Laboratory of Agricultural Microbiology, College of Veterinary Medicine, Huazhong Agricultural University, Shizishan Street, Wuhan, 430070, China

## Abstract

**Background:**

Metal ions are important micronutrients in cellular metabolism, but excess ions that cause toxic reactive oxygen species are harmful to cells. In bacteria, Fur family proteins such as Fur, Zur and PerR manage the iron and zinc uptake and oxidative stress responses, respectively. The single Fur-like protein (annotated as PerR) in *Streptococcus suis* has been demonstrated to be involved in zinc and iron uptake in previous studies, but the reports on oxidative stress response and gene regulation are limited.

**Results:**

In the present study, the *perR* gene deletion mutant Δ*perR* was constructed in *Streptococcus suis* serotype 2 strain SC-19, and the mutant strain Δ*perR* exhibited less sensitivity to H_2_O_2_ stress compared to the wild-type. The *dpr* and *metQIN* were found to be upregulated in the Δ*perR* strain compared with SC-19. Electrophoretic mobility shift assays showed that the promoters of *dpr* and *metQIN* could be bound by the PerR protein. These results suggest that *dpr* and *metQIN* are members of the PerR regulon of *S. suis*. *dpr* encodes a Dps-like peroxide resistance protein, and the *dpr* knockout strains (Δ*dpr* and Δ*dpr*Δ*perR*) were highly sensitive to H_2_O_2_. MetQIN is a methionine transporter, and the increased utilization of methionine in the Δ*perR* strain indirectly affected the peroxide resistance. Using a promoter–EGFP gene fusion reporting system, we found that the PerR regulon was induced by H_2_O_2_, and the induction was modulated by metal ions. Finally, we found that the pathogenicity of the *perR* mutant was attenuated and easily cleared by mice.

**Conclusions:**

These data strongly suggest that the Fur-like protein PerR directly regulates *dpr* and *metQIN* and plays a crucial role in oxidative stress response in *S. suis*.

## Background

Iron and zinc are recognized as important micronutrients for bacteria, but excess of iron can catalyze the Fenton reactions, resulting in formation of toxic hydroxyl radicals [1]. Similarly, an excess of zinc ions can also trigger the formation of hydroxyl radicals [2]. Besides hydroxyl radicals, reactive oxygen species (ROS) such as superoxide radical and H_2_O_2_ are inevitably generated as byproducts of aerobic metabolism in bacteria [3]. Additionally, during infection, ROS can be generated by the innate immune system[4]. ROS can cause damage to many macromolecules including DNA, proteins and lipids [5,6]. It is clear that oxidative stress and metal homeostasis are closely related. However, bacteria have evolved efficient mechanisms to maintain metal ion homeostasis and protect themselves from oxidative damage [7].

Fur family proteins are present widely in bacteria and play crucial roles in cellular processes. This family contains more than six different proteins. They are the sensors of iron (Fur and Irr) [8][9], zinc (Zur) [10], manganese [11] and nickel (Nur) [12], and the peroxide regulon repressor (PerR) [13]. In the Gram-negative *Escherichia coli*, there are two Fur family proteins Fur and Zur. In contrast, there are three Fur-like proteins (Fur, Zur and PerR) in many Gram-positive bacteria such as *Bacillus subtilisClostridium acetobutylicum* and *Staphylococcus aureus.* In *B. subtilis*, Fur regulates iron uptake and siderophore biosynthesis; Zur regulates two ABC zinc transporters; and PerR regulates the oxidative stress response [13,14].

*Streptococcus suis* is economically a very important Gram-positive and facultative anaerobic bacterium that causes severe diseases in pigs and humans. As an emerging zoonotic pathogen, *S. suis* serotype 2 has become the predominant causative agent of adult human meningitis in Vietnam and Hong Kong [15]. Two large outbreaks of human infections were reported in China in 1998 and 2005, resulting in 229 infections and 52 deaths [16,17]. Like other bacterial pathogens, *S. suis* may also encounter both oxidative stress and metal starvation during infection. Thus, the regulation on the responses to oxidative stress and metal starvation by Fur-like proteins could be particularly important for *S. suis* survival *in vivo* and pathogenesis. However, only a single gene encoding a Fur-like protein has been found in each sequenced genome of *S. suis*, even in the genomes of most species of the genus *Streptococcus*. For example, the single Fur-like protein is encoded by SSU05_0310 in *S. suis* serotype 2 strain 05ZYH33 (GenBank accession no. CP000407). This protein has been defined as a zinc uptake regulator (Zur) [18], as well as an iron uptake regulator (Fur) in *S. suis*[19], but the research on its function in oxidative stress response is limited, whereas its homolog in *Streptococcus pyogenes* has been demonstrated to be a peroxide regulon repressor PerR [20-22]. In this study, the role of this Fur-like protein in peroxide resistance was confirmed in *S. suis* serotype 2. Therefore, we renamed this protein as PerR. At the same time, two target operons, *dpr* (*dps*-like peroxide resistance protein) and *metNIQ* (methionine ABC-type transporter), were identified and proved to play important roles in oxidative stress response.

## Results

### Identification of a fur-like protein in *S. Suis* and other streptococci

**Figure 1:**
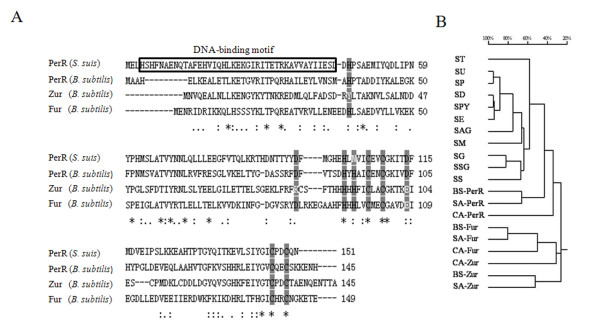
**Fur-like proteins are conserved among the genus*Streptococcus*and are close to PerR.** (A) Multiple alignment of PerR protein from *S. suis* 05ZYH33 with the Fur family proteins PerR, Zur and Fur in *B. subtilis* str. 168. The DNA-binding motif is marked in the gray box. Nine conserved amino acid residues in PerR are marked with gray bottom colour. Five residues (H37, D85, H91, H93 and D104) are the candidate amino acid ligands for Fe^2+^ or Mn^2+^ and four cysteine residues (C96, C99, C136 and C139) are for Zn^2+^, H37 and H91 are the sites of H_2_O_2_-mediated oxidation. These amino acid residues in *S. suis* PerR protein are conserved except that N is taking the place of H in site 93, this change also exists in *S. pyogenes.* (B) A phylogenetic tree of Fur-like proteins from selected streptococci and other Gram-positive bacteria was constructed based on a multiple sequence alignment using DNAMAN. Fur-like proteins in each streptococcus are represented by the abbreviation of strain name. BS, *B. subtilis* 168, CA, *C. acetobutylicum* ATCC 824, SA, *S. aureus* Mu50, SAG, *S. agalactiae* 2603 V/R, SD, *S. dysgalactiae* GGS_124, SE, *S. equi* MGCS10565, SG, *S. gordonii* CH1, SM, *S. mutans* NN2025, SP, *S. parauberis* KCTC 11537, SPY, *S. pyogenes* M1 GAS, SS, *S. suis* 05ZYH33, SSG, *S. sanguinis* SK36, ST, *S. thermophilus* CNRZ1066, SU, *S. uberis* 0140 J.

In the genome of 05ZYH33 (a strain of *S. suis* serotype 2), the Fur-like protein encoded by SSU05_0310 had been first identified as a Zur [18], and we found that SSU05_0310 is the sole gene encoding a Fur-like protein in *S. suis* 05ZYH33. The SSU05_0310 protein consisted of 151 amino acids and contained a DNA-binding motif (Figure 1A). To identify the Fur-like proteins in other streptococci, a BLAST homology search using the sequence of SSU05_0310 was performed among the sequenced genomes of the members of genus *Streptococcus*. All streptococci had a single conserved Fur-like protein except that no Fur-like protein was found in *Streptococcus pneumoniae*. All the Fur-like proteins in streptococci and their homologs (Fur, Zur and PerR) in *B. subtilisS. aureus* and *C*. *acetobutylicum* were used for cluster analysis, the result showed that the Fur-like proteins in streptococci clustered in the PerR group (Figure 1B). Furthermore, through sequence analysis, the key amino acid residues of PerR for H_2_O_2_ response and metal ions binding were highly conserved in SSU05_0310 protein (Figure 1A) [23]. Consequently, we named the single Fur-like protein in *S. suis* as PerR.

### Roles of PerR in H_2_O_2_ resistance in *S. Suis*

Our sequence analysis suggested that PerR might be involved in the oxidative stress response in *S. suis*, and therefore we constructed a *perR* knockout strain (Δ*perR*) and a functional complementing strain (CΔ*perR*). The growth of the wild-type, mutant and complementary strains showed no obvious difference in TSB medium with 5% newborn bovine serum (data not shown).

**Figure 2:**
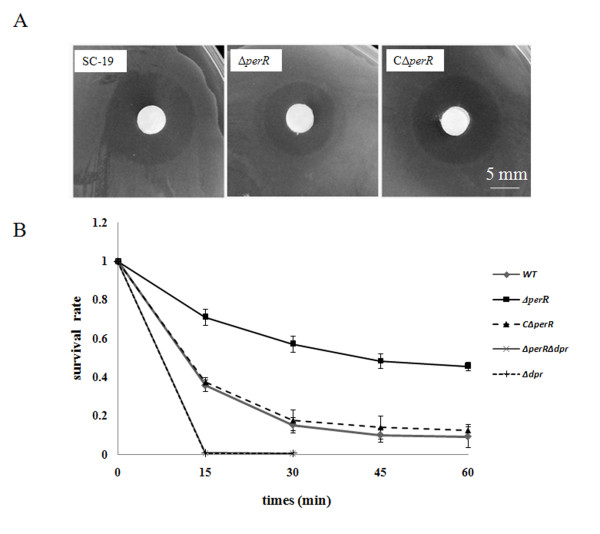
***S. suis*sensitivity to peroxide stress.** (A) The H_2_O_2_ sensibility was tested by disk diffusion assay. 1 M H_2_O_2_ was used. (B) The survival rates of wild-type (WT), Δ*perR*, CΔ*perR*, Δ*dpr* and Δ*perR*Δ*dpr* at every 15 min in TSB with 10 mM of H_2_O_2_ challenge. Three independent experiments were performed.

To characterize the roles of *perR* in the susceptibility of *S. suis* to peroxide stress, the sensitivity of the wild-type strain SC-19, mutant strain Δ*perR* and complementing strain CΔ*perR* to H_2_O_2_ was compared using an inhibition zone assay. As shown in Figure 2A, the strains SC-19 and CΔ*perR* (about 16.3 mm and 16.1 mm in diameter) exhibited larger inhibition zones than the Δ*perR* strain (about 12.7 mm in diameter) when 4 μl of 1 M H_2_O_2_ was used. To determine further the difference in H_2_O_2_ sensitivity, quantitative analysis was performed. As shown in Figure 2B, after H_2_O_2_ (10 mM) treatment, the *perR* mutant strain showed a higher survival rate than the wild type. The survival rate of the complementary strain CΔ*perR* was similar to that of the wild-type strain. These results indicated that inactivating *S. suis perR* led to reduced sensitivity to H_2_O_2_.

### Transcriptional regulation by PerR in *S. Suis*

**Figure 3:**
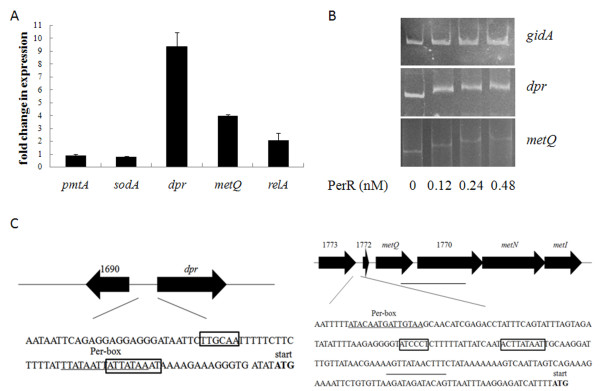
**Identification of PerR regulon in*S. suis.*** (A) Relative expression levels of genes *dpr*, *metQ*, *relA*, *pmtA* and *sodA* in strain Δ*perR* compared to its parental strain SC-19. Relative abundance of the transcripts was determined by real-time RT-PCR from the total RNAs derived from strains Δ*perR* and SC-19 in mid-log phase. *gapdh* was used as the internal control. (B) Different concentration of PerR proteins binds to *dpr* and *metQIN* promoters (500 bp and 300 bp respectively), *gidA* promoter (300 bp) was used as the negative control. (C) The structure of the *dpr* and *metQIN* promoters. -10 and −35 regions of the promoters are shown by the boxes. The start codon is labeled by blod fonts. The predicted PerR-box is underlined.

PerR has been recognized as an important regulator in bacteria. In order to identify members of the PerR regulon in *S. suis*, according to the consensus sequence of the PerR-box in *S. pyogenes* and *B. subtilis* (NTANAANNATTNTAN) [21,22], we screened for putative PerR-boxes in the −500 to +50 sequences of all the genes/operons in the *S. suis* 05ZYH33 genome. 12 predicted binding sites and 19 supposed target genes and operons were identified. The transcriptional levels of all 19 supposed target genes and operons (including *dprmetQrelA* and *pmtA*) containing prospective PerR-box in the promoters were compared between the strains SC-19 and Δ*perR* by real-time RT-PCR (Table 1). Only three genes *dpr* (Dps-like peroxide resistance protein), *relA* (GTP pyrophosphokinase) and *metQ* (methionine transporter) were significantly upregulated (≥two-fold) in Δ*perR* (Figure 3A). Electrophoretic mobility shift assay (EMSA) showed that the His-tagged recombinant PerR protein could bind to the promoters of *dpr* and *metQIN*, but not to those of *relApmtA* and *gidA* (*gidA* was used as the negative control, the results of *relA* and *pmtA* were not shown) (Figure 3B). These results suggest that the *dpr* gene and *metQIN* operon were directly regulated by PerR. The PerR boxes in the promoters of *dpr* and *metQIN* are shown in Figure 3C. To confirm regulation by PerR in *S. suis*, a transcriptional reporter plasmid pSET4s:P_dpr_*-*EGFP was inserted into the genomes of strains SC-19 and Δ*perR*. When cultured in TSB with 5% newborn bovine serum, stronger green fluorescence was observed in strain Δ*perR*:EGFP compared to SC-19:EGFP by fluorescence microscopy. The mean fluorescence intensity (MFI) was measured by flow cytometry (MFI of Δ*perR*:EGFP: 56.85 ± 1.015, MFI of SC-19:EGFP: 25.29 ± 1.965).

**Table 1:** The results of PerR regulon’s identification

**Predicted target genes**^**a**^	**Gene names**	**Function of genes**	**Predicted PerR-box** **NTANAANNATTNTAN**	**qRT-PCR**^**b**^	**EMSA results**
SSU05_0022		aromatic amino acid aminotransferase	ATAAAACTATTATAA	−2.5 (0.6)	
SSU05_0209		hypothetical protein	CTATAATCATTTTAT	+1.1 (0.2)	
SSU05_0308		hypothetical protein	GTAAAATTATTATAA	−1.1 (0.1)	
SSU05_0309	*pmtA*	cation transport ATPase	TTAGAATTATTATAA TTATAACGATTATAA	−1.1 (0.1)	negative
SSU05_0618		MATE efflux family protein	TTAAAATAATTATAA	−4.2 (1.1)	
SSU05_1264		SAM-dependent methyltransferase	ATAGAATTATTATAA	−1.1 (0.3)	
SSU05_1265		sulfatase	ATAGAATTATTATAA	−1.8 (0.3)	
SSU05_1341	*lacI*	LacI family transcriptional regulator	TTAGAATCATTCTAG	−1.8 (0.4)	
SSU05_1689	*dpr*	peroxide resistance protein	TTATAATTATTATAA	+9.3 (1.1)	positive
SSU05_1691		phosphotyrosine protein phosphatase	TTATAATTATTATAA	−1.7 (0.4)	
SSU05_1771	*metQ*	lipoprotein transporter	ATACAATGATTGTAA	+4.0 (0.2)	positive
SSU05_1855	*escA*	ABC transporter ATP-binding protein	ATATAATTATTATAA	−16.1 (5.2)	
SSU05_1856		HIT-family protein	ATATAATTATTATAA	−1.6 (0.4)	
SSU05_2094	*relA*	GTP pyrophosphokinase	GTATAATGATTGTAG	+2.1 (0.6)	negative
SSU05_2095	*cpdB*	2',3'-cyclic-nucleotide 2'-phosphodiesterase	GTATAATGATTGTAG	−3.0 (1.1)	
SSU05_2112		hypothetical protein	GTATAATGATTATAC	−1.5 (0.6)	
SSU05_2113	*rarA*	recombination factor protein	GTATAATGATTATAC	+1.7 (0.5)	
SSU05_2191	*rlmH*	rRNA large subunit methyltransferase	ATAAAATAATTGTAA	−1.3 (0.3)	
SSU05_2192	*htrA*	trypsin-like serine protease	ATAAAATAATTGTAA	+1.2 (0.3)	

^a^*S. suis* ORF number of *S. suis* 05ZYH33

^b^Fold-change (standard deviation) of expression in Δ*perR* compared to expression in wild-type

**Figure 4:**
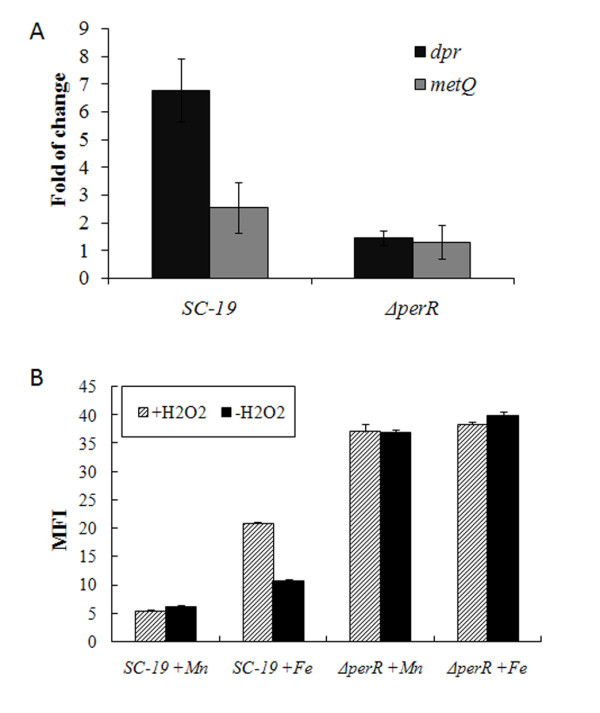
**H**_**2**_**O**_**2**_**and metal ions affect the expression of the PerR regulon.** (A) Relative transcript levels of *dpr* and *metQIN* after 10 μM H_2_O_2_ stimulating. (B) Expression of EGFP in strains SC-19 and Δ*perR* in the CDM supplemented with different metal ions. The cells were grown to mid-log phase in the basal CDM with 50 μM Zn^2+^ and 50 μM Fe^2+^ or Mn^2+^ and treated with or without 10 μM H_2_O_2_ 4 times in every 15 min. The final mean fluorescence intensity (MFI) was calculated by each sample’s MFI deducting the MFI of negative control (no EGFP inserted SC-19).

The effects of H_2_O_2_ on the transcriptional regulation were tested. Bacteria were stimulated by 10 μM H_2_O_2_ for 10 min, the expression levels of *dpr* and *metQIN* were analyzed by qRT-PCR. As shown in Figure 4A, *dpr* and *metQIN* was obviously induced in SC-19 but not in Δ*perR* (cultured in TSB). Then, the EGFP reporter strains were used, the MFI of strains SC-19:EGFP and Δ*perR*:EGFP in chemical defined medium (CDM) was measured. As shown in Figure 4B, for the strain SC-19:EGFP, growth in medium with 50 μM zinc and 50 μM manganese led to a low green fluorescence level, and no obvious induction by H_2_O_2_ (10 μM) could be detected. In contrast, when grown in medium with 50 μM zinc and 50 μM iron, SC-19:EGFP expressed a relatively high level of EGFP, and the MFI was about two-fold higher after induction by H_2_O_2_ for 1 h. The MFI of strain Δ*perR*:EGFP was high and had no significant change in each condition. These results suggest that PerR regulated the target operons by binding to the promoter region, and the derepression was induced by H_2_O_2_ and influenced by metal ions.

### Roles of *dpr* in H_2_O_2_ resistance in *S. Suis*

H_2_O_2_ sensitivity analysis suggested that PerR was involved in oxidative stress response and we have found that *dpr* was directly regulated by PerR in *S. suis*. *dpr* encodes a peroxide resistance protein, previous study has found that *dpr* mutant was highly sensitive to H_2_O_2_[24]. To test the role of *dpr* in H_2_O_2_ resistance, the *dpr* gene was inactivated in strains SC-19 and Δ*perR*. The resultant mutant strains Δ*dpr* and Δ*perR*Δ*dpr* were subjected to the H_2_O_2_ sensitivity assay. Both *dpr* mutant strains exhibited <1% survival after incubation with 10 mM H_2_O_2_ (Figure 2B). Inactivation of *dpr* led to near loss of H_2_O_2_ defensive capability in both Δ*dpr* and Δ*perR*Δ*dpr* strains. However, there was no obvious difference in the survival rate between Δ*dpr* and Δ*perR*Δ*dpr*, suggesting that the increased H_2_O_2_ resistance of the *perR* mutant probably results of the derepression of *dpr*.

### Role of methionine in H_2_O_2_ resistance in *S. Suis*

**Figure 5:**
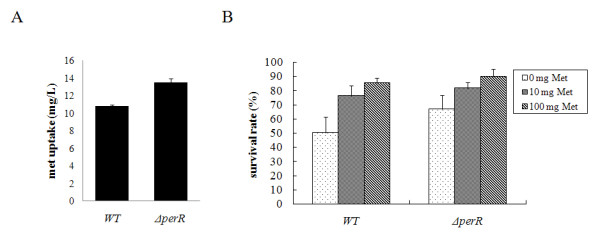
**Roles of methionine in the H**_**2**_**O**_**2**_**resistance.** (A) The amount of uptaken methionine in the wild type (WT) and Δ*perR* in cells grown to late-log phase. (B) The effects of the methionine to H_2_O_2_ resistance. Survival rates of wild-type (WT) and Δ*perR* in CDM with 5 mM of H_2_O_2_ challenge for 30 min. 0, 10 and 100 mg/l of methionine were added in the methionine-free basal CDM respectively.

Expression of the methionine ABC transporter *metQIN* was upregulated in the Δ*perR*, therefore, methionine uptake may have been increased in the mutant. To verify this hypothesis, the methionine utilization by strains SC-19 and Δ*perR* was investigated by measuring the reduced amount of methionine in the CDM. There was no obvious different in the growth rate of strains SC-19 and Δ*perR*, but the amount of methionine utilization in the mutant was increased by 25.13% compared to the wild type in cells grown to late-log phase (Figure 5A). These data indicated that the derepression of *metQIN* led to increased accumulation of methionine in strain Δ*perR*.

To investigate the role of methionine in oxidative stress, the H_2_O_2_ sensitivity of strains in CDM with different concentrations of methionine was tested. As shown in Figure 5B, strain SC-19 showed the lowest survival rate in CDM lacking methionine, and the survival rates were increased when methionine was added. The same phenomenon was observed in strain Δ*perR*, except that Δ*perR* showed higher survival rates at every methionine concentration. These results indicated that the resistance to H_2_O_2_ in *S. suis* was related to methionine.

### Role of PerR in pathogenicity in *S. Suis*

An experimental infection model in mice was designed to assess the role of PerR in pathogenicity. In the wild-type group, all of the mice presented severe clinical signs associated with septicemia and septic shock during the first day post-infection and then died from septicemia in this group. In contrast, the mice in the Δ*perR* group presented with partial clinical signs, three of eight infected mice survived during 1 dpi, and finally one mouse was alive at 7 dpi. Thus, as previously report [25], the mutant strain Δ*perR* was slightly attenuated in pathogenicity according to survival rate and clinical signs.

To investigate the reason of the reduced pathogenicity in *perR* mutant, mice were intraperitoneally infected with the same dose of SC-19 and Δ*perR*. Bacteria were recovered from blood, lung, brain and spleen. At 7 dpi, the numbers of Δ*perR* harvested from blood and each tissue were significantly decreased compared to those of the wild-type strain. At 11 dpi, the Δ*perR* was nearly cleared from mice, but the wild-type strain could still be recovered (Table 2). Statistical significance of the difference was determined by student *t*-test. The result suggested that the viability of *perR* mutant was reduced in the host.

**Table 2:** Survival of SC-19 and Δ*perR* in different organs in mice

**Source**	**Strain**	**Bacteria recovered from blood and tissues (×10**^**5**^** CFU)**^**a**^		
		**4 dpi**	**7 dpi**^**b**^	**11 dpi**^**b**^
Blood	SC-19	4.49 ± 3.24	2.37 ± 1.71	0.44 ± 0.04
	**Δ***perR*	4.10 ± 2.41	0.09 ± 0.05	0
Lung	SC-19	4.22 ± 1.45	1.48 ± 0.11	1.03 ± 1.59
	**Δ***perR*	1.66 ± 1.11	0.07 ± 0.04	0
Brain	SC-19	5.07 ± 3.07	1.42 ± 0.20	1.62 ± 1.33
	**Δ***perR*	3.84 ± 2.96	0.13 ± 0.12	0.01 ± 0.01
Spleen	SC-19	0.15 ± 0.09	0.35 ± 0.11	0.03 ± 0.02
	**Δ***perR*	0.22 ± 0.22	0.04 ± 0.04	0

^a^ Mean ± standard deviation of 4 independent experiments. Date is expressed as CFU/ml blood, or CFU per tissue.

^b^*P*<0.05 for comparison of SC-19 versus Δ*perR* CFU at 7 and 11 dpi (student’s *t*-test).

## Discussion

As a pathogen, *S. suis* may encounter both oxidative stress and metal starvation during infection. Fur family proteins play important roles in metal ion homeostasis and oxidative stress responses in many bacteria. A single Fur-like protein was identified in *S. suis*, and in the rest of the genus *Streptococcus*, except for *S*. *pneumoniae*. The Fur-like protein in *S. suis* has been shown to regulate the zinc and iron uptake genes [18,19]. In our study, the function of this Fur-like protein in oxidative stress response was characterized. We suggested that, in addition to its role in regulating zinc and iron uptakes, another important role of this Fur-like protein was to act as an oxidative stress response regulator in *S. suis*, and reannotated this Fur-like protein as PerR.

A recent research has found that the *fur* (*perR*) knock-out mutant in *S. suis* serotype 2 strain P1/7 was more sensitive to H_2_O_2_[25]. However, in our study, an opposite result was observed, that deletion of *perR* in *S. suis* serotype 2 strain SC-19 resulted in increased resistance to H_2_O_2_. Deletion of PerR has been found to cause a high resistance ability to H_2_O_2_ in *B. subtilis*[13], *C. acetobutylicum*[26]*S. aureus*[27], and in the single Fur containing *S. pyogenes*[21], and these results accord with our test in *S. suis*.

As a negative regulator, the high resistance to H_2_O_2_ in *perR* mutant may result from derepression of the PerR regulon. In many bacteria, one important member of PerR regulon for H_2_O_2_ resistance is catalase [28]. However, all lactic acid bacteria including *S. suis* lack catalase, it is interesting to identify other potential PerR targets for H_2_O_2_ resistance in *S. suis*. qRT-PCR and EMSA tests showed that *dpr* and *metQIN* were directly regulated by PerR, and the expression of *dpr* and *metQIN* could be induced rapidly by physiological level of H_2_O_2_. These results suggested that one mechanism for oxidative stress response by PerR was derepression of PerR targets *dpr* and *metQIN*. Previous study found that *feoAB* was regulated by Fur (reannotated as PerR in our study) in *S. suis* P1/7 strain [19], however, in our study the PerR protein could not bind with *feoAB* promoter as well as we did not found a PerR-box in the promoter region (data not shown), suggesting that it is an indirectly regulation.

Dps family proteins have been identified in many bacteria including *S. suis*. In *B. subtilis* and *S. pyogenes*, the Dps homolog MrgA is derepressed when H_2_O_2_ oxidizes PerR [21,29]. Usually, If the Fe^2+^ is present, H_2_O_2_ could be nonenzymatically cleaved into highly toxic hydroxyl radicals by Fenton reaction (H_2_O_2_ + Fe^2+^ → ^·^OH + ^―^OH + Fe^3+^). However, Dpr can prevent the Fenton-reaction by storing iron and converting Fe^2+^ to Fe^3+^-mineral (FeOOH) in a ferroxidase dependent way, resulting in avoiding formation of hydroxyl radicals. In addition, Dpr can bind DNA to protect DNA from oxidative damage in most bacteria but not in *S. suis*[30-32]. According with previous study, H_2_O_2_ resistance was markedly reduced in Δ*dpr*[24]. In our experiment, we found that the double mutant Δ*perR*Δ*dpr* was also highly sensitive to H_2_O_2_ (Figure 2B). Although other PerR targets might be derepressed in Δ*perR*, H_2_O_2_ resistance ability was not obviously increased. It suggested that, in catalase negative *S. suis*, Dpr was especially crucial for H_2_O_2_ resistance, and the main reason for increased H_2_O_2_ resistance in Δ*perR* was derepression of *dpr*.

All amino acid residues of protein are susceptible to oxidative stress. However, methionine sulfoxide can be reduced to methionine by methionine sulfoxide reductase (Msr). During this reaction, Methionine helps the organisms to reduce H_2_O_2_ to H_2_O (Met + H_2_O_2_ → Met(O) + H_2_O; Met(O) + Th(SH)_2_ → Met + Th(S-S) + H_2_O) [33]. In most species, such as humans, mice, yeast and bacteria, the cyclic oxidation and reduction of methionine residue plays an important role in defense against oxidative stress [33-36]. In our study, the *metNIQ* operon was found to be regulated by PerR. However, the *metNIQ* operon is repressed via the S-box system in *B. subtilis* and in some other bacteria [37]. In contrast, we did not find the S-box in the promoter of *metNIQ* operon in *S. suis*, but it was replaced by a PerR-box (Figure 3C). A recent report also found that *metNIQ* operon was regulated by PerR in *S. pyogenes* via microarray assay [38]. It seems, that *metQIN* is negatively regulated by Fur-like protein, is special in the streptococci. We found that *metQIN* operon could be induced by H_2_O_2_ in SC-19, and in *metQIN* derepressed Δ*perR*, methionine utilization was increased. Additionally, methionine concentration was found to be related to H_2_O_2_ resistance. These results suggested that, via controlling the methionine transport, methionine uptake could be regulated by PerR. Thus, oxidative stress response was indirectly affected.

**Figure 6:**
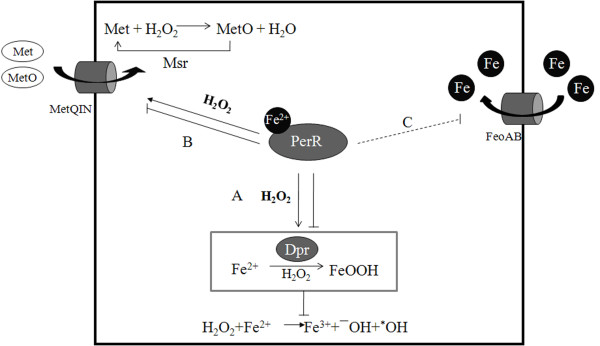
**Schematic presentation of the PerR regulatory oxidative stress response in*S. suis*.** (A) *dpr* is repressed by PerR, and derepression of *dpr* could be induced by H_2_O_2_. Abundant Dpr stores iron to prevent Fenton reaction. (B) derepression of *metQIN* is induced by H_2_O_2_, leading to increasing Met (methionine) and MetO (methionine sulfoxide) uptake. During Met cyclic oxidation and reduction, H_2_O_2_ can be reduced to H_2_O. (C) FeoAB is negatively regulated by PerR. (The broken lines indicate that the regulatory mechanisms were unclear).

Metal ions level played an important role in oxidative stress response, especially iron level. In our study, using the transcriptional reporter system, we found that PerR represses the regulon by binding to the promoters, and derepression of the regulon could be induced by H_2_O_2_ when abundant Fe^2+^ was added. In *B. subtilis*, the regulatory mechanism of PerR has been well studied from the standpoint of its structure, revealing that PerR is a dimeric zinc protein with a regulatory site that coordinates either Fe^2+^ or Mn^2+^. PerR can bind Fe^2+^ or Mn^2+^ and then repress transcription of its targets, however Fe^2+^ can catalyze the oxidation of key histidine in PerR, leading to inactivation of PerR [23,39]. PerR in *S. suis* may have a similar regulatory mechanism to that of *B. subtilis* PerR. According to our results and previous studies, we summarized the putative PerR mediated oxidative stress response pathway in *S. suis* and showed it in Figure 6.

PerR has been found to be necessary for full virulence of *S. pyogenes*[20]. Our investigation found that the pathogenicity of *perR* mutant strain was attenuated. The decreased pathogenicity might be due to the reduced viability of mutant in the host. The fact that the viable number of mutant recovered from mice was much less than that of the wild-type, also supported this explanation. It seems that deletion of *perR* may lead to inappropriate expression of PerR-regulated genes and affect the normal growth. For example, knockout of *perR* led to iron starvation and the growth was inhibited *in B. subtilis*[28]. It was reported that, because Dpr could store iron, the cytosolic iron would be efficiently scavenged when *dpr* was ectopic overexpressing in *S. suis*[31]. It suggested that in Δ*perR*, the derepressed *dpr* would lead to cytosolic iron starvation and affect the growth.

## Conclusions

These data strongly suggest that the Fur-like protein PerR regulates the oxidative stress response in *S. suis*. Two members of PerR regulon *dpr* and *metQIN* were identified in *S. suis*, *dpr* played a crucial role in H_2_O_2_ resistance and *metQIN* might indirectly affect the H_2_O_2_ resistance by controlling the methionine uptake. Mice infection model showed that the pathogenicity of *perR* mutant strain was attenuated.

## Methods

### Bacterial strains, plasmids, and growth conditions

All the bacterial strains and plasmids used in this study are listed in Table 3. *S. suis* serotype 2 strain SC-19 was isolated from diseased pigs in Sichuan province, China in 2005 [40]. *S. suis* was grown in tryptic soy broth (TSB) or on tryptic soy agar (TSA; Difco, Detroit, MI, USA) plates containing 5% newborn bovine serum (Sijiqing, Hangzhou, China). The CDM [41], modified when necessary, was also used to culture *S. suis*. *E. coli* strains DH5α and BL21 (DE3) were cultured in/on Luria–Bertani broth or plates (Oxoid, Basingstoke, UK). When necessary, antibiotics were added to the plates or broth at the following concentrations: 100 μg/ml spectinomycin (Spc), 2.5 μg/ml erythromycin (Erm) or 5 μg/ml chloramphenicol for *S. suis*; 50 μg/ml Spc, 180 μg/ml Erm, 12.5 μg/ml Chl or 50 μg/ml kanamycin [22][22] for *E. coli*.

**Table 3:** Strains and plasmids used in this study

**Strains or plasmids**	**Characteristics**	**Reference or source**
Strains		
SC-19	Virulent Chinese *S. suis* serotype 2 isolate, wild-type	This work
**Δ***perR*	Gene *perR* inactive strain, Erm^r^	This work
C**Δ***perR*	Complemented **Δ***perR* strain, Erm^r^ Spc^r^	This work
**Δ***dpr*	Gene *dpr* inactive strain, Spc^r^	This work
**Δ***perR*Δ*dpr*	Gene *perR* and *dpr* inactive strain, Erm^r^ Spc^r^	This work
SC-19:EGFP	P_dpr_-EGFP fusion inserted Wild-type, Spc^r^	This work
**Δ***perR*:EGFP	P_dpr_-EGFP fusion inserted **Δ***perR*, Spc^r^	This work
DH5αand BL21(DE3)	Cloning and expression host	In this lab
Plasmids		
pSET4s	Thermosensitive allelic replacement vector	[42]
pSET2	*E. coli*-*S. suis* shuttle vector	[44]
pET28a	His tag fusion expression vector	Novagen
pMIDG310	A plasmid containing a EGFP gene	
pSET4s:: *perR*	A mosaic plasmid designed to inactivate *perR*	This work
pSET4s::*dpr*	A mosaic plasmid designed to inactivate *dpr*	This work
pSET2::C*perR*	Recombinant plasmid used for functional complementation of Δ*perR*	This work
pSET4s:EGFP	Recombinant plasmid used for inserting a P_dpr_-EGFP fusion	This work
pET28a:: *perR*	Recombinant expression plasmid to produce His_6_-fused PerR protein	This work
pAT18	A plasmid containing an *erm*	[45]

### Expression and purification of the PerR protein

The whole coding sequence of *perR* was amplified from the genomic DNA of *S. suis* SC-19 using primers 310 F/310R (Table 4), which were designed according to the SSU05_0310 sequence of *S. suis* 05ZYH33 (GenBank accession no. CP000407), and cloned into a prokaryotic expression vector pET-28a (+) (Novagen, Shanghai, China). The resultant plasmid pET28a::*perR* was confirmed by DNA sequencing and transformed into *E. coli* BL21 (DE3) for expression of His-tagged recombinant protein PerR induced by 1 mM isopropyl-beta-D-thiogalactopyranoside at 18°C for 4 h. Purification of the recombinant protein was achieved using Ni-NTA agarose (Bio-Rad. USA) under native conditions according to the manufacturer’s instructions. Electrophoresis was carried out with 12% SDS-PAGE.

**Table 4:** Primers used in this study

**Primers**	**Sequence**	**Restriction site**	**target**
General PCR amplification			
310 F	CGTACAGTCGACTTAGTTCTGGCAATCAGGACA	*Sal*I	*perR*
310R	CGTATCGGATCCATGGAACTCCATTCTCACTTC	*Bam*HI	
310 L01:	TAGTAAGCTTCACAGTTGGACCTTGGTT	*Hind* III	Left arm of *perR*
310 L02	TCACCTGCAGCGGCATTTGTCCTGATTG	*Pst*I	
310R01	TCACCTGCAGTTAGCATTGAAGTGAGAATGG	*Pst*I	Right arm of *perR*
310R02	AGGTGAATTCTTGCTACTGTAATGGTCG	*Eco*RI	
ermF	TCACCTGCAGGAGTGTGTTGATAGTGCA	*Pst*I	*erm*^*r*^
ermR	AGGTCTGCAGCTTGGAAGCTGTCAGTAG	*Pst*I	
C310F	TCACCTGCAGGATGATGTGGCTGTGTTG	*Pst*I	*perR* and its promoter
C310R	TAGTGGATCCAAGTCATGTCCGTCGTAG	*Bam*HI	
PdprF	TCAGAATTCTCGGGCTATAGGTAAAAG	*Eco*RI	Promoter of *dpr*
PdprR	TCAGGATCCATATCACCCTTTCTTTTATT	*Bam*HI	
EGFP 01	TCAGGATCCATGAGTAAAGGAGAAGAAC	*Bam*HI	EGFP gene
EGFP 02	TCACTGCAGTGCTATTTGTATAGTTCATC	*Pst*I	
1772P01	TCCAGGACTGGTGGCGAC		Promoter of 1772
1772P02	AAAATGATCTCCTTAAATTA		
relAP01	CATATCTCTACTCTTCCTC		Promoter of *relA*
relAP02	AGCTAGTGTGAGTGCTAC		
gidAP01	CATGTTGTTCTCTCCTTC		Promoter of *gidA*
gidAP02	TTGAGGTCAATGAGGTAG		
Real-time RT-PCR			
0309 F	GCAACACTTTCTGCCATCA		*pmtA*
0309R	GGTCGCACCTACAACTTCA		
1771 F	CGCACCAATCCGTCTTTA		*metQ*
1771R	TTTCGTTTGTTGGGTCGT		
2094 F	TAAGACCGACGAATCCC		*relA*
2094R	TCATCCGCGACAGCT		
1689 F	TTTATCAGTAGCCCATTCA		*dpr*
1689R	AAACGCTCACTCATCTCA		
1539 F	AGAAGGCAAGTTGGAAG		*sodA*
1539R	GTAGTTTGGACGGACATT		
0155 F	AGAAGTAAACGCTGCTAT		*gapdh*
0155R	CAAACAATGAACCGAAT		

### Construction of strains

To knockout the gene *perR* from *S. suis* SC-19, a thermosensitive homologous suicide vector pSET4s::*perR* carrying the left arm, right arm and the Erm resistance cassette (*erm*^r^) was constructed. The two arms were amplified from the chromosomal DNA of SC-19 by using primers 310 L01/310 L02 and 310R01/310R02 (Table 4), respectively. The *erm*^r^ was amplified from the plasmid pAT18 by using primers ermF/ermR (Table 4). The recombinant plasmid pSET4s::*perR* was electrotransformed into SC-19, and the strains were selected on Spc and Erm plates as described previously [42]. The suspected mutant strain Δ*perR* was verified by PCR, RT-PCR and Southern blot analysis. To construct a functional complementary strain for Δ*perR*, the complete coding sequencing of *perR* with its upstream promoter was amplified and cloned into the *E. coliS. suis* shuttle vector pSET2. The resultant plasmid pSET2::*perR* was electrotransformed into the mutant strain Δ*perR*. The resultant complementary strain was designated as CΔ*perR*.

To monitor the regulation to *dpr* promoter, pSET4s:P_dpr_*-*EGFP, a thermosensitive plasmid containing the transcriptional reporter system was constructed as follow: a 500-bp fragment containing the *dpr* promoter was amplified from SC-19 genomic DNA using primers PdprF/PdprR and cloned between the *Eco*RI and *Bam*HI sites of the plasmid pSET4s, resulting in a plasmid pSET4s:P_dpr_. The EGFP gene coding sequence was amplified from pMIDG301 (kindly donated by Dr Paul Langford, London, UK) using primers EGFP01/EGFP02 and cloned between the *Bam*HI and *Pst*I sites of the plasmid pSET4s:P_dpr_. The resultant plasmid pSET4s:P_dpr_-EGFP was electrotransformed into *S. suis* SC-19 and Δ*perR*, respectively. The fragment containing the *dpr* promoter was used as the homologous arm, through a single cross event, the thermosensitive plasmid pSET4s:P_dpr_-EGFP was inserted into the genome at 28°C and the rest of plasmids in the strains were lost for continuous passage culture at 37°C. Spc was used in the whole process. The resultant strains were confirmed by PCR.

### GFP assays

The CDM lacking zinc, iron and manganese was used as the basal medium. Overnight cultured *S. suis* strains SC-19:EGFP and Δ*perR*:EGFP were washed three times using the basal CDM, and then diluted 1:100 in the basal CDM supplemented with 50 μM Zn^2+^ and Fe^2+^ (or Mn^2+^) and 50 μg/ml Spc. Cells were cultured at 37°C for 3–4 h to early mid-log phase (OD_600_ = 0.3). The cells were induced by 10 μM H_2_O_2_ four times at every 15 min. One hour later, 1 ml of each sample was obtained and washed with PBS three times, green fluorescence was observed by fluorescence microscopy, and the mean fluorescence intensity (MFI) was assayed by flow cytometry. To remove the background of green fluorescence, strain SC-19 was used as the negative control.

### H_2_O_2_ sensitivity assays

The disk diffusion assay to test H_2_O_2_ sensitivity was performed as described previously [43]. The strain was cultured under near-anaerobic conditions to mid-log phase and 100-μl aliquots were spread on TSA plates. A sterile 5-mm-diameter filter disk containing 4 μl 1 M H_2_O_2_ was placed on the surface of the TSA plate. After incubation at 37°C for 12 h, the size of the area cleared of bacteria (inhibition zone) was measured.

For quantitative analysis, resistance of *S. suis* to H_2_O_2_ killing was tested as described previously [20], with slight modifications. Overnight cultured bacteria were diluted 100-fold into fresh TSB containing 5% newborn bovine serum in sealed tubes at 37°C without shaking (near-anaerobic conditions). When OD_600_ of the cells reached ~0.5, some cells were removed and incubation was continued at 37°C without agitation, and 10 mM H_2_O_2_ was added to the other part of the bacterial culture. Samples were collected at every 15 min for 1 hour after addition of H_2_O_2_. Appropriate bacterial dilutions were plated on TSA plates for viability counts. Survival rate was calculated by dividing the number of CFUs in the H_2_O_2_ challenge part with the number in the part without H_2_O_2_ challenge. For testing the effect of methionine on H_2_O_2_ resistance, overnight cultured bacteria were diluted 100-fold in CDM with different concentrations of methionine and then tested as above.

### Amino acid analysis

Overnight cultured bacteria were washed three times with CDM and resuspended in the medium containing 100 mg/l methionine (OD_600_ = 0.1), and then incubated at 37°C for ~4 h. When the growth of cultures reached the late-log phase (OD_600_ = 1.6), medium samples were withdrawn from the bioreactor directly into a 2-ml tube. Samples were filtered through 0.22-μm filters. Amino acid concentrations of the filtered samples were determined using Amino Acid Analyzer L-8900 (Hitachi, Tokyo, Japan). All standards were commercial amino acids (Ajinomoto, Japan).

### Electrophoretic mobility shift assay (EMSA)

Binding of recombinant PerR protein to DNA fragments containing the putative PerR-box was performed. The DNA fragments of the candidate promoters were amplified from *S. suis* SC-19 genomic DNA and purified by using the PCR Product Purification Kit (Sangon Biotech, Shanghai, China). Binding reactions were carried out in a 20-μl volume containing the binding buffer (20 mM Tris–HCl, pH 8.0; 50 mM KCl; 5% glycerol; 0.5 mM DTT; 25 μg/ml BSA, 100 ng poly dIdC), 0.1 μg promoter DNA and different amounts of purified recombinant PerR protein (0, 2, 4, and 8 μg). Binding reaction was incubated at room temperature for 15 min. The loading buffer was then added to the reaction mixtures and the electrophoresis was carried out with 5% native polyacrylamide DNA retardation gels at 100 V for ~1 h. Finally, the gels were stained with ethidium bromide. The 300-bp promoter of *gidA* was used as negative control.

### Real-time RT-PCR

Total RNAs of *S. suis* strains SC-19 and Δ*perR* were isolated as follows: overnight cultured bacteria in TSB medium with 5% newborn bovine serum was diluted 1:100 in fresh serum-containing TSB, and then incubated at 37°C to the mid-log phase (OD_600_ = 0.5). Total RNA was isolated and purified using the SV Total RNA Isolation System (Promega) according to the manufacturer’s instructions. The contaminating DNA was removed by DNase I treatment. Transcripts of the target genes were assessed by real-time RT-PCR using SYBR Green detection (TAKARA. Dalian. China) in an ABI 7500 system. *gapdh* gene served as the internal control. The primers using in the real-time RT-PCR are listed in Table 4. Differences in relative transcript abundance level were calculated using the 2^–ΔΔCT^ method.

### Mouse model of infection

All animal experiments were carried out according to the Regulation for Biomedical Research Involving Animals in China (1988). To detect the role of PerR in virulence in *S. suis*, a total of 24 female 6-week-old Balb/C mice were divided into three groups (8 mice per group). Animals in groups 1 and 2 were inoculated by intraperitoneal injection with 1 ml ~6.125 × 10^7^ CFU of either *S. suis* SC-19 or Δ*perR* diluted in TSB. TSB medium was used as a negative control for group 3. Mice were observed for 1 week. To detect the role of FzpR PerR in colonization, two groups of female 6-week-old Balb/C mice were inoculated by intraperitoneal injection with 1 ml of 5 × 10^7^ CFU of either SC-19 or Δ*perR* diluted in physiological saline. Blood, brain, lung and spleen were collected from mice (4 mice in each group) at 4, 7 and 11 days post infection (dpi). The samples were homogenized and subjected for bacterial viability count on TSA plates.

## Authors’ contributions

TZ participated in the design of study, performance of the experiments and the writing of manuscript. YD, TL and YW participated in the performance of the experiments. WL participated in the design of the study. RZ and HC participated in the design of study and the writing of manuscript. All authors read and approved the final manuscript.
